# The Immunomodulatory Function of Assembled Composite Nanopolypeptide Containing Bursal-Derived BP7 (CNPB7) in Promoting the Mucosal Immune Response within Poultry Immunization

**DOI:** 10.3390/vaccines12080834

**Published:** 2024-07-24

**Authors:** Xinyu Guo, Jianing Hu, Guihu Yin, Yiqin Cai, Zichen Gao, Ye Liu, Meng Zhong, Ruiying Wang, Xiuli Feng

**Affiliations:** 1 Key Laboratory of Animal Microbiology of China’s Ministry of Agriculture, College of Veterinary Medicine, Nanjing Agricultural University, Nanjing 210095, China; 2022807117@stu.njau.edu.cn (X.G.);; 2 MOE Joint International Research Laboratory of Animal Health and Food Safety, College of Veterinary Medicine, Nanjing Agricultural University, Nanjing 210095, China

**Keywords:** polypeptide self-assembly, composite nanoparticles CNPB7, non-specific immune response, mucosal immune response, antibody production

## Abstract

Mucosal immunity is the main defense line against respiratory disease pathogens. Newcastle disease and avian infectious bronchitis are common respiratory diseases in poultry. However, the mucosal immune response is not sufficiently activated and thus fails to achieve the ideal immune protection. Therefore, it is important to develop a suitable mucosal immune adjuvant to enhance the immune response of live vaccines. Here, the bursal-derived peptide BP7, β-glucan, and hyaluronic acid were selected as the adjuvant to be assembled into the composite nanopolypeptide adjuvant (CNPB7) with ultrasonic dispersion technology. The results showed that after optimizing assembly conditions, the optimal average particle size of nanoparticle CNPB7 was 514.9 nm and PDI was 0.298. To evaluate the non-specific immune responses of nanoparticle CNPB7, the chickens were immunized only with nanoparticle CNPB7. It was confirmed that nanoparticle CNPB7 enhanced the expression of CD3, CD4, CD80, and CD86 factors in the spleen lymphocyte from the chicken immunized with nanoparticle CNPB7. To investigate the mucosal immune response of nanoparticle CNPB7, the chickens were orally immunized with Newcastle disease virus (NDV)–infectious bronchitis virus (IBV) dual vaccines and CNPB7. The results proved that the levels of immunoglobulin SIgA, IL-4, IFN-γ, and IL-13 in the mucus samples from the respiratory and digestive tract in chicken immunized with nanoparticle CNPB7 and vaccines were significantly increased, compared to that of vaccine control. Finally, it was observed that nanoparticle CNPB7 promoted specific increased antibody productions against NDV and IBV in the immunized chicken. These results proved that the assembled nanoparticle CNPB7 could enhance the vaccination efficacy in chicken, which provided the experimental basis for the development of new adjuvants, and offered technical support for preventing virus transmission of avian diseases.

## 1. Introduction

Due to the prevalence of clinical diseases, vaccine immunization has become a major prevention and control measure, in which adjuvants are becoming an increasingly important topic of concern [1,2]. Newcastle disease and avian infectious bronchitis are common respiratory diseases in poultry [3,4], and mucosal immunity is the main defense line against respiratory disease pathogens [5,6]. However, traditional vaccines often induce weaker mucosal immune responses, with the limited duration, and the immune tolerance induced by frequent vaccination. The current vaccination strategies against NDV and IBV are relatively unsatisfactory. Therefore, it is crucial to design and develop a suitable mucosal immune adjuvant to enhance the immune response of live vaccines. Considering the selection requirements for oral vaccine adjuvants and the advantages of nanoparticle materials as immune effectors and antigen carriers, nanoparticle becomes an adjuvant improvement strategy to improve the mucosal immune efficiency of oral vaccine administration [7], which may help improve the immune efficacy of vaccines against Newcastle disease and avian infectious bronchitis.

The bursa of Fabricius (BF) is currently recognized as a central humoral immune organ, and is the key site for B cell differentiation and maturation. BP7, a bioactive peptide derived from BF, is isolated and identified as a polypeptide molecule that promotes immune response [8]. In the immune model of the avian influenza virus (AIV) antigen, BP7 mainly activates the Th1 type immune response and plays a role in the Th2 type immune response. BP7 is also associated with multiple immune-related biological processes, demonstrating the multifunctional inducibility in humoral and cellular immune responses [8,9]. It is worth noting that BP7 can also induce autophagy in immature B cells. Furthermore, BP7 activates B cell differentiation, and induces numerous differentially expressed genes in chicken macrophage HD11 cells [10]. These results consolidate the potential of BP7 as an effective immune adjuvant for AIV vaccines. As a natural polysaccharide adjuvant, β-glucan has good immune regulatory functions on vaccine immunization [11]. Also, hyaluronic acid, as a transdermal absorbent, is often studied as a potential drug delivery carrier and can also promote the absorption of nutrients [12]. These above-mentioned three biomaterials have become potential alternatives for developing new immune adjuvants.

An immune adjuvant is one of the key components in vaccine formulations, which contributes to the immune process of vaccination, thereby enhancing and prolonging the recognition and memory of vaccine antigens in the immune system [13]. An immune adjuvant can promote antigen presentation and activate various immune cells (such as T cells, B cells, and dendritic cells), leading to the intercellular communication enhancement and the production of cytokines and chemical factors, which ultimately leads to stronger and more persistent antibody responses and the formation of memory cells [14].

Mucosal immunity plays a crucial role in protecting the body from pathogen invasion. However, the mucosal immune response induced by live vaccines is usually weak and short-lived. The oral vaccination is widely used in the NDV-IBV combined vaccine, which can stimulate the mucosal immune system of the respiratory system in chickens. Considering the selection requirements for oral vaccine adjuvants and the advantages of nano/particle materials as immune effectors and antigen carriers [14,15,16], the composite nanopolypeptide adjuvant is the novel candidate adjuvant to improve the efficiency of oral vaccine administration.

In order to investigate the immune induction function of new nanoparticle adjuvants on mucosal immune response, in this study, the composite polypeptide nanoparticle adjuvant was constructed with active peptide BP7, β-glucan, and hyaluronic acid, which was named as CNPB7. The extracellular stimulation and chicken immunization experiments were performed to evaluate the potential immune functions of CNPB7. Furthermore, the ten-day-old chicken was immunized with CNPB7 and NDV-IBV dual vaccine to explore the immune efficacy on the specific immune responses and mucosal immune responses. The study will provide experimental evidence for the development of new adjuvants and technical support for preventing poultry respiratory diseases.

## 2. Materials and Methods

### 2.1. Peptide, and Cells

BP7 (GGCDGAA) was synthesized by Sangon Biotech (Shanghai, China) with a purity of 90% and identified by high-performance liquid chromatography (HPLC) and mass spectrometry (MS) analysis.

DF-1 cells and Vero cells were preserved in the Infectious Disease Laboratory, the School of Animal Medicine, Nanjing Agricultural University, which were cultured with Dulbecco’s modified eagle medium (DMEM) medium with 10% fetal bovine serum (FBS) at 37 °C and 5% CO_2_.

### 2.2. MTT Assay

DF-1 cells were treated with BP7, β-glucan, and hyaluronic acid from 5 μg/mL to 500 μg/mL, respectively, in which BP7, β-glucan, and hyaluronic acid were dissolved in a phosphate-buffered saline (PBS) buffer, in which PBS was used as control and DMEM medium was used as mock control. After 24 h, 20 μL 3-(4,5)-dimethylthiahiazo (-z-y1)-3,5-diphenytetrazoliumromide (MTT) was added into DF-1 cells for 4 h. The culture medium was discarded, and 150 μL dimethyl sulfoxide (DMSO) reagent was added to the cells. The absorbance at optical density (OD)_570_ nm was measured on an enzyme-linked immunosorbent assay (ELISA) analyzer, and the cell viability was represented as stimulation index (SI) according to the following formula.

SI = (OD_570_ experimental group/OD_570_ control group) × 100%

### 2.3. Preparation and Optimization of Composite Nanoparticle CNPB7

According to the reported methods [17,18], the ultrasound dispersion technology was employed for the self-assembly process of peptides, by which BP7, β-glucan, and hyaluronic acid were assembled into the composite polypeptide nanoparticles. Simply, β-glucan was dissolved in PBS solution containing 1% (*w*/*v*) Tween 80 to be 250 μg/mL, and hyaluronic acid was dissolved in PBST containing 1% (*w*/*v*) sodium tripolyphosphate to a final concentration of 500 μg/mL. Then, β-glucan, hyaluronic acid, and BP7 at different concentrations were mixed under magnetic stirring for 4 h, and the mixture was dispersed in an equal volume of water by an ultrasonic crusher under an ice bath for 5 min. Finally, during the pH value of the solution, 0.1% acetic acid was added for adjustment for further steps.

Additionally, the chemical modification was carried out by adding sodium tripolyphosphate and sodium hydroxide solutions to change the type of crosslinking agent. Also, the different magnetic stirring times (2–4 h) and different surfactants Tween 20 and Tween 80 were regulated to optimize the particle size, dispersion coefficient polydispersity index (PDI), and zeta potential of the nanoparticles. The aim of optimization was to obtain a moderately sized and stable system of composite nanoparticles for subsequent experiments.

### 2.4. Nanoparticle Size and Potential Determination of Nanoparticle CNPB7

In this experiment, the hydrodynamic diameter and PDI of nanoparticles were measured with a nanoparticle potential meter Mastersizer 2000 (Malvern Panalytical technologies: Malvern, UK) [19]. The parameters were listed as follows. The measurement angle was set at 90 degrees, the temperature was controlled at 25 °C, and the waiting time was 2 min (120 s). The determination of the zeta potential was achieved, and the specific data of particle size were obtained through a nanoparticle potential meter. Additionally, the nanoparticle potential meter was used to determine the average particle size, PDI, and zeta potential of the nanoparticle samples. The test sample was diluted with water at 1:100 before measurement. Then, the sample was placed in a plastic colorimetric dish for testing to record the relevant data of average particle size, PDI, and zeta potential.

### 2.5. Transmission Electron Microscopy (TEM) of Nanoparticle CNPB7

TEM was conducted according to the reported method [20]. Firstly, the nanoparticle samples were diluted with deionized water at 1:100. After being dropped onto a copper mesh, the nanoparticle samples were negatively stained with 2% phosphotungstic acid solution for 2 min. Subsequently, the excess dye was removed and the copper mesh was air-dried naturally in an indoor environment. Finally, the size and morphological characteristics of the nanoparticles were examined and recorded using a transmission electron microscope (H-7650, Hitachi, Tokyo, Japan).

### 2.6. Cell Counting Kit-8 (CCK8) Assay

The effects of nanoparticle CNPB7 on the viabilities of DF-1 cells were detected using the CCK8 method [21]. DF-1 cells were treated with 50, 100, and 200 μg/mL of nanoparticle CNPB7 for 24 h. BPS was used as a control. CCK-8 solution was added into DF-1 cells with 10 μL per well for 4 h. After shaking for 10 min, the absorbance of DF-1 cells was measured at 450 nm using an ELISA reader (ELx800, Bio-Tek, Winooski, VT, USA).

### 2.7. The Nonspecific Innate Immune of Chicken Immunized with Nonopartical CNPB7

#### 2.7.1. Chicken Immunization Only with Nanoparticle CNPB7

Ten twenty-day-old Hyline brown chickens purchased from Nanjing Tegeili Planting Professional farm (Nanjing, China) were randomly divided into two groups. One group of chickens was orally immunized once with 100 μg/mL nanoparticles containing BP7. The other group of chickens was orally immunized with a BPS solution and was used as a control.

#### 2.7.2. Immune Organ Index Detection of the Chicken Immunized with Nanoparticle CNPB7

On the seventh day after the immunization, the spleen and bursa were collected from the immunized chicken to measure the immune organ indicators. According to the reported calculation method [22], immune organ index = weight of immune organs (g)/body weight (kg).

#### 2.7.3. Immune Factors Detection of the Chicken Immunized with Nanoparticle CNPB7

Then, the spleen lymphocytes were collected from the immunized chicken on the seventh day after immunization, and the mRNA expressions of cytokines CD3, CD4, CD80, and CD86 were detected using the RT-PCR method, in which the primer sequences designed were listed in Table 1. The related primer sequences were designed based on the sequences of CD3 (NC_052555.1), CD4 (NC_052532.1), CD80 (NC_052532.1), and CD86 (NC_052532.1), and β-actin (NC_052545.1) was used as the reference gene.

### 2.8. The Specific Innate Immune of Chicken Immunized with Nanopartical CNPB7 and NDV-IBV Dual Vaccine

#### 2.8.1. Vaccines Immunization Groups

Fifty ten-old-day Hyline brown chickens were randomly divided into five groups. Three groups of chickens were immunized with the mixture of the NDV-IBV dual vaccine and 50, 100, and 200 μg nanoparticle CNPB7 /chicken. Also, the chickens of the same breed and age were immunized with the NDV-IBV vaccine and PBS as the vaccine and negative controls, respectively. All experimental group chickens were orally immunized once.

#### 2.8.2. The Viabilities of Spleen Lymphocytes from the Immunized Chicken

On the fourteenth day after vaccine immunization, the spleen lymphocytes were isolated from the immunized chicken, according to the instructions of the chicken spleen lymphocyte separation kit. The lymphocytes were cultured with 1640 medium containing 10% FBS, and treated with 10 ng/mL lipopolysaccharide (LPS) for 48 h. Then, 10 μL CCK8 solution was added to lymphocytes for 4 h, and the absorbance was detected at 450 nm. The lymphocyte viabilities were represented as stimulation index.

#### 2.8.3. ELISA Detection for sIgA, Cytokines, and IBV Antibody Productions

On the fourteenth day after immunization, the respiratory and digestive mucus samples were collected, and the levels of secretory immunoglobulin A (sIgA), IL-4, and IFN-γ were detected and analyzed, according to the corresponding ELISA kit procedures.

Also, the antibody levels of IBV from the immunized chicken on the fourteenth day after the immunization were measured with the indirect ELISA method [23]. Simply, 6 μg/mL recombinant S1 protein of IBV was coated on ELISA plates at 4 °C overnight. After being blocked with PBST containing 5% defatted milk powder, the sera collected from the immunized chicken was added to an ELISA plate, and then horseradish peroxidase (HRP)-labeled rabbit anti-chicken secondary antibody (SA00001-6, proteintech, Wuhan, China) was added at 1:10,000 dilution ratio. After washing, the chromogenic substrate solution was added for 10 min, and then 2 M H_2_SO_4_ was added for the termination of the color reaction. The absorbance was detected at 450 nm, and the antibody production was analyzed and compared.

#### 2.8.4. Fluorescence Quantitative PCR for IL-13

The total RNAs from the intestinal mucosal tissue were extracted with Trizol following the procedure as the reported method [24]. The mRNA expressions of IL-13 were detected with a qPCR kit (PR036A, Takara, Shiga, Japan), following the kit manual. Primers were designed based on the sequences of IL-13 (GU119894.1) from the online NCBI database (Table 1), in which β-actin (NC_052545.1) was used as the reference gene. The reaction procedure of qPCR with the involved primers is shown in Table 2.

#### 2.8.5. Virus Neutralization Test for NDV Antibody

The neutralizing antibody levels of NDV in the serum of immunized chickens were identified using a neutralization assay with fixed virus dilution serum [25]. Simply, the sera were collected from all immunized chickens on the fourteenth day after immunization. The sera were diluted from 2^1^ to 2^8^, and mixed with 100 tissue culture infective dose (TCID)_50_/0.1 mL NDV for 1 h. Then, Vero cells were incubated with mixture containing the immunized sera and NDV, and cultured with a minimum essential medium (MEM) containing 2% FBS for 120 h. Based on the (cytopathic effect, CPE), 50% of the serum neutralization endpoint protection dose (PD)_50_ was calculated.

### 2.9. Data Analysis

In this study, Graphpad Prism 5.0 software was used to perform *t*-test analysis of the experimental data. The significant differences between data were statistically analyzed, and the magnitude of these differences was calculated and represented as the mean ± standard deviation (SD). The significance difference is marked as * *p* < 0.05, ** *p* < 0.01, and *** *p* < 0.001.

## 3. Results

### 3.1. No Cytotoxicity of BP7, β-Glucan, and Hyaluronic Acid

**Figure 1:**
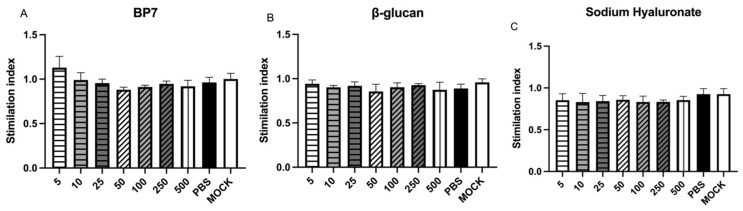
Effects of different concentrations of BP7, β-glucan, and hyaluronic acid on the cytotoxicity of DF-1 cells. DF-1 cells were treated with BP7, β-glucan, and hyaluronic acid, and the viabilities were detected with MTT assay. (**A**) BP7; (**B**) β-glucan; (**C**) hyaluronic acid.

To investigate the cytotoxicity of BP7, β-glucan, and hyaluronic acid, the cell viabilities of DF-1 were measured following MTT assay. As shown in Figure 1, there were no significant differences in the activity of DF-1 cells after BP7 treatment at a range of 5 μg/mL to 500 μg/mL (Figure 1A). Also, the viabilities of DF-1 cells treated with β-glucan and hyaluronic acid at the experimental concentrations were similar to PBS control. These results suggested that BP7, β-glucan, and hyaluronic acid might not possess cytotoxicity, and possess good biocompatibility.

### 3.2. Determination and Characterization of Optimal Nanoparticles

The nanoparticles were optimized through changes in the system of crosslinking agents, magnetic stirring time, and different surfactants. The obtained optimal parameter is shown in Table 3.

It was found that the optimal parameter of nanoparticles was 1% sodium tripolyphosphate, 1% Tween 80, and magnetic stirring 4 h. The optimal particle size of composite nanoparticles CNPB7 was 514 nm, with a PDI of 0.298.

**Figure 2:**
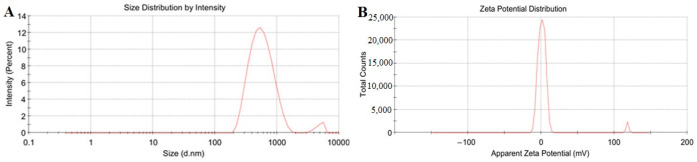
Hydrodynamic dimensions and zeta potential of nanoparticles CNPB7. (**A**) The size distribution by intensity. (**B**) The zeta potential distribution.

Furthermore, the particle size and zeta potential distribution in the hydrated state of the optimal composite nanoparticles CNPB7 were measured using a nanoparticle potential analyzer, as shown in Figure 2. It was observed that the hydrodynamic size of CNPB7 presented the normal distribution, and showed a single narrow distribution peak (Figure 2A). Also, the distribution map of the zeta potential of CNPB7 showed a single narrow distribution peak, with the highest peak value around 25 mV (Figure 2B), whose potential value was 26.5 Mv with negative charge (Table 3). The results indicate that the prepared composite nanoparticles CNPB7 might have the expected size, with the characteristics of uniformly dispersed, stable, high potential, and a stable system.

**Figure 3:**
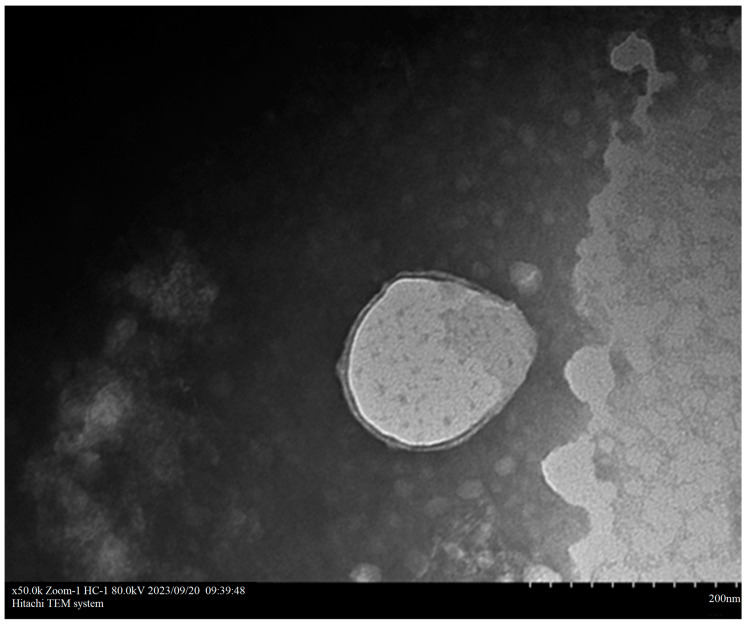
Transmission electron microscopy images of nanoparticle CNPB7.

The imaging results of TEM showed that the nanoparticle CNPB7 exhibited an elliptical shape, with a relatively uniform particle size distribution and no aggregation phenomenon (Figure 3). Additionally, the encapsulation of peptide nanoparticles by β-glucan and hyaluronic could be clearly observed, which further confirms the measurement results of hydrodynamic particle size and potential data (Figure 2).

### 3.3. Nanoparticle CNPB7 Had No Cytotoxicity on DF-1 Cells

**Figure 4:**
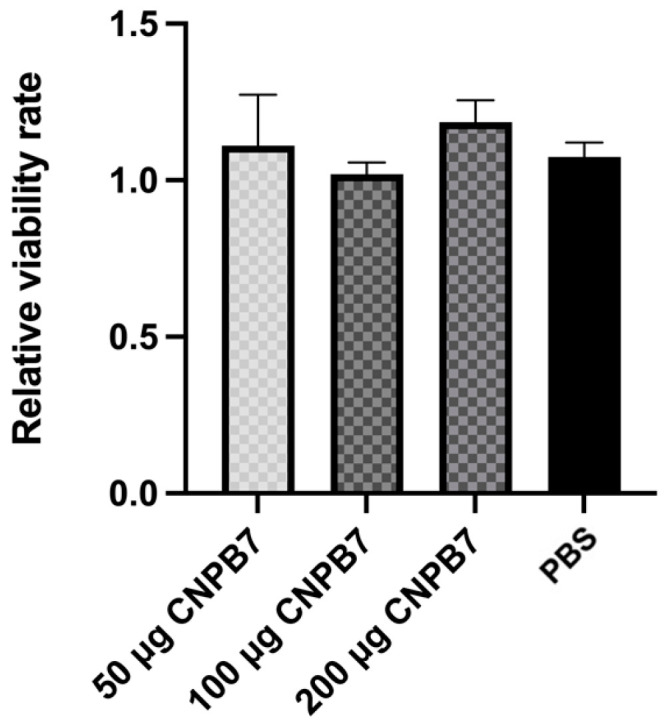
The cytotoxic effect of CNPB7 on viabilities of DF-1 cells. DF-1 cells were treated with CNPB7 at three concentrations for 24 h, and the viabilities were measured with MTT assay.

The CCK8 method was used to detect the effect of nanoparticle CNPB7 on the activity of DF-1 cells in vitro. The results showed that the viabilities of DF-1 cells treated with CNPB7 were not significantly different from that of the PBS control (Figure 4), suggesting that the nanoparticle CNPB7 has no cytotoxicity on DF-1 cells.

### 3.4. Nanoparticle CNPB7 Promoted Non-Specific Immune Response in Chickens

**Figure 5:**
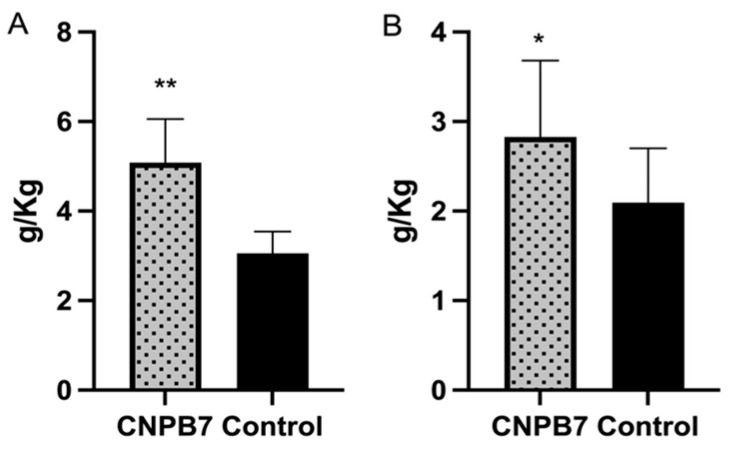
The effect of nanoparticle CNPB7 on bursa and spleen of the chicken. Five chickens were orally immunized with nanoparticle CNPB7 for seven days, and five chickens were orally immunized with PBS as control (*n* = 5). Then, the bursa and spleen of the immunized chicken were measured. (**A**) Bursa index. (**B**) Spleen index. * *p* < 0.05, ** *p* < 0.01, compared to that of control.

To investigate the inducing role of the nanoparticle on non-specific immune response in chickens, the bursa and spleen of the chicken orally immunized with nanoparticle CNPB7 were analyzed. The results showed that the spleen index of chicken immunized with 100 μg/mL nanoparticles CNPB7 was 2.83 (Figure 5A), and the bursa index was 5.08 (Figure 5B), while the spleen index of PBS control was 2.09, and the bursa index was 3.06. These results proved that the immune organ indexes of the spleen and bursa in the nanoparticles CNPB7 group were significantly increased, compared to the control.

**Figure 6:**
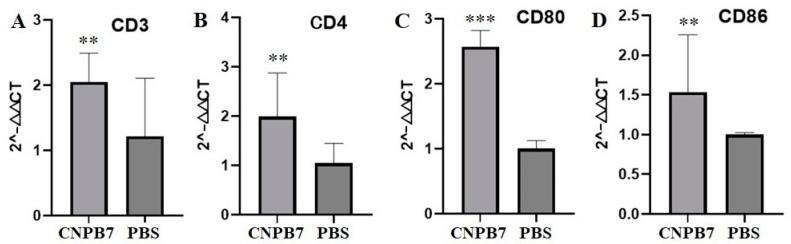
The inducing roles of nanoparticle CNPB7 on immune-related factors. Five chickens were orally immunized with nanoparticle CNPB7, and five chicken were orally immunized with PBS as control (*n* = 5). The mRNAs of four factors in spleen lymphocytes from the immunized chicken were detected on the 7th day after immunization. (**A**) CD3. (**B**) CD4. (**C**) CD80. (**D**) CD86. ** *p* < 0.01, *** *p* < 0.001, compared to that of PBS control.

Furthermore, it was observed that the expressions of surface factor CD3 and CD4 of T cells in the spleen from the chicken immunized with nanoparticle CNPB7 were significantly increased, compared to that of PBS control (Figure 6A,B). Also, the expressions of CD80 and CD86 in the CNPB7-immunized chicken were significantly higher than that of PBS control (Figure 6C,D). These results suggested that nanoparticle CNPB7 could activate various immune-related factors in chickens.

### 3.5. Nanoparticle CNPB7 Induced Various Biological Factor Expressions during Vaccination

**Figure 7:**
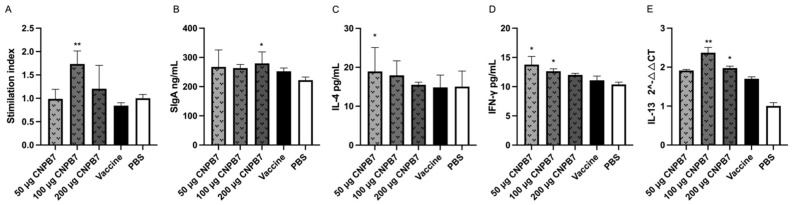
The function of nanoparticle CNPB7 on lymphocyte viabilities and immune factors in chicken immunized with vaccines. Chickens were orally immunized with vaccine and three concentrations of nanoparticle CNPB7 (*n* = 10). On the 14th day after immunization, the spleen lymphocyte viabilities were measured with CCK8 assay. The mucus samples of respiratory and digestive tract from the immunized chicken were collected to detect the levels of sIgA, IL-4 and IFN-γ following ELISA kit, and expression of IL-13 was measured using qPCR. (**A**) The viabilities of lymphocytes. (**B**) sIgA production. (**C**) IL-4 production. (**D**) IFN-γ production. (**E**) IL-13 expression. * *p* < 0.05, and ** *p* < 0.01, compared to vaccine control.

To investigate the function of nanoparticle CNPB7 on lymphocyte viabilities, the spleen lymphocytes were isolated from the chicken immunized nanoparticle CNPB7 and vaccine on the fourteenth day after immunization, and was detected with CCK8 method. It was observed that the lymphocyte viabilities of the experimental group with 100 and 200 μg nanoparticles CNPB7 were higher than that of vaccine control, in which 100 μg nanoparticles CNPB7 were highest among all the experimental groups (Figure 7A). Additionally, the cell viability of 50 μg CNPB7 was similar to that of the live vaccine control. These results suggested that the composite nanoparticles CNPB7 should have no cytotoxicity on cell viabilities.

In order to verify the adjuvant effect of nanoparticle CNPB7 on the mucosa immune of chicken, sIgA, IL-4, and IFN–γ in the mucus samples from the respiratory and digestive system were detected on the fourteenth day after immunization. It was observed that compared to that of vaccine control, the secretion levels of SIgA from the experimental group immunized with nanoparticle CNPB7 and vaccine were increased, in which 200 μg nanoparticle CNPB7 induced the highest sIgA production (Figure 7B).

Also, the IL-4 secretion levels in chicken immunized with nanoparticle CNPB7 with three concentrations were higher than those in the vaccine control group (Figure 7C), in which 50 μg CNPB7 induced the highest IL-4 production among all groups (*p* < 0.05).

Furthermore, the levels of IFN-γ secretion in the experimental group with three concentrations of nanoparticle CNPB7 were higher than that of vaccine control (Figure 7D), in which the levels of IFN-γ in 50 and 100 μg nanoparticle CNPB7 immunization were significantly increased. Additionally, the expression levels of IL-13 in the nanoparticle CNPB7 group with different concentrations of immunization were higher than that of the vaccine group, in which 100 μg nanoparticle CNPB7 induced the highest IL-13 expression (Figure 7E). These results suggested that nanoparticle CNPB7 might induce the functional factors response in the mucosal immune system.

### 3.6. Nanoparticle CNPB7 Induced Specific Antibody Production during Vaccination

**Figure 8:**
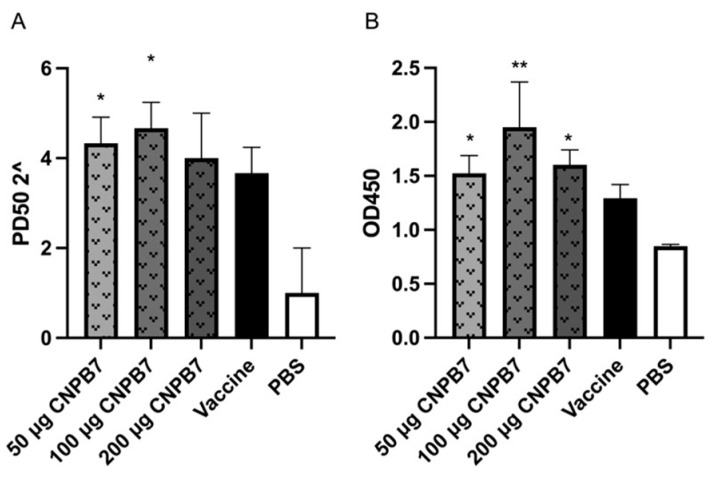
The antibody levels with nanoparticle CNPB7 immunization. On the 14th day after immunization, the antibody levels of NDV in the immunized chicken (*n* = 10) were detected with a neutralizing experiment, and IBV antibody productions were measured with the ELISA method. (**A**) NDV antibody levels. (**B**) IBV antibody levels. * *p* < 0.05, and ** *p* < 0.01, compared to vaccine control.

The sera samples were collected from the immunized chicken on the fourteenth day after immunization to detect the antibody levels of NDV and IBV. The neutralizing antibody level of NDV is shown in Figure 8A. It was observed that the neutralizing antibody levels of chicken immunized with vaccine and nanoparticle CNPB7 at three concentrations were higher than that of vaccine control, in which antibody levels from the 100 μg nanoparticle CNPB7 groups were highest among all the experimental groups. Furthermore, the results of ELISA showed that the OD values of three concentrations of nanoparticle CNPB7 in the experimental group were significantly higher than those in the vaccine control, and the highest effect was observed in 100 μg nanoparticle CNPB7 group among all groups on the fourteenth days after immunization (Figure 8B). The above results indicated that nanoparticle CNPB7 might induce the humoral immune response, and increase the antibody levels of vaccine immunization.

## 4. Discussion

Newcastle disease and infectious bronchitis are clinically common and important diseases affecting the respiratory system of poultry [3,4]. The mucosal immune response will be of great significance for the prevention and control of these two diseases. Vaccination through the mucosal tissue is the most effective way to trigger a protective mucosal immune response [7,14,15]. Nanoparticle adjuvants and drug delivery systems can improve the immunogenicity of antigens and induce stronger mucosal immune responses [16,17]. Therefore, safe and effective vaccine adjuvants and drug delivery systems are important strategies to promote the mucosal vaccines.

### 4.1. Characterization of Nanoparticle CNPB7

In this paper, the composite polypeptide nanoparticle CNPB7 was constructed with active peptide BP7, β-glucan, and hyaluronic acid, based on peptide self-assembly technology. Polymer nanoparticles and microparticles have been proven to have enormous potential as drug-delivery systems [19]. Especially, biodegradable polymer nanoparticles are used to embed antigens such as proteins, peptides, or DNA, which can control the release of vaccine antigens and optimize the required immune response by selectively targeting antigen-presenting cells [26,27]. Peptide copolymers can self-assemble into various aggregates, so peptides can be combined with active substances to prepare the nanoparticle adjuvants. Furthermore, BP7, β-glucan, and hyaluronic acid were proven to not be cytotoxic. It was crucial to determine the concentration range of the biomaterials used in the experiment through MTT experiments, since the cytotoxicity experiments help evaluate the clinical doses. The biocompatibilities of BP7, β-glucan, and hyaluronic acid provide guidance for determining the concentration of nanoparticle adjuvants to be used in subsequent animal immune experiments.

The literature indicates that in order to meet the nanometer level, the average diameter of nanoparticles needs to be within a certain range, and the particle size distribution should be evaluated through PDI values, in which a PDI value of less than 0.3 indicates good dispersion [19]. Also, the high surface charge, i.e., an absolute value of zeta potential greater than 30 mV, indicates the strong interparticle repulsion, which is beneficial for the stability of the nanoparticle in solution [28]. In this paper, the peptide assembly conditions of nanoparticles were optimized based on the concentration of sodium tripolyphosphate, the type of surfactant, pH, and ultrasound conditions. Performance and quality control of nanoparticles were evaluated following the zeta potential, particle size, and PDI analysis. The satisfactory nanoparticle CNPB7 was obtained, whose average particle size was 514.9 nm, and PDI was 0.298, which could be used for subsequent animal immunization experiments.

### 4.2. The Effect of Nanoparticle CNPB7 on Non-Specific Immune Function in Chickens

Adjuvants can activate the immune system and guide strong immune responses [29]. Oral nanoparticle adjuvants promote non-specific immune responses in chickens, which is of great significance for new adjuvants development of oral and spray vaccine. In this paper, nanoparticle CNPB7 significantly enhanced the functional indicators of immune organs such as spleen and BF, indicating the potential application of nanoparticle CNPB7 as adjuvants for animal vaccines. Furthermore, nanoparticle CNPB7 promoted the expression of molecules such as CD3, CD4, CD80, and CD86 in splenic lymphocytes. CD3 is a marker molecule on the surface of mature T cells, which is the key component of T cell antigen recognition and signal transduction [30,31]. CD4^+^T cells help B cells to produce antibodies, promote macrophage bactericidal ability, and guide CD8^+^T cells to develop into effector cells [32,33]. The increased level of CD3 and CD4 factors indicated an increase in T cell activation and/or proliferation, suggesting that the cellular immune system might be activated under the action of adjuvant, which could induce the non-specific immune responses in vivo.

CD80 (also known as B7-1) and CD86 (also known as B7-2) are co-stimulatory molecules located on the surface of antigen-presenting cells, and play key roles in T cell activation and immune response [34,35]. During the activation process of T cells, it is necessary to the co-stimulatory signals, in addition to recognizing antigen presentation complexes (MHC antigen peptide complexes) through T cell receptors (TCRs) [36,37]. Therefore, the increased levels of CD80 and CD86 in spleen lymphocytes from the chicken immunized with nanoparticle CNPB7 were the important markers of adjuvant-induced T cell-mediated immune response, which indicated the activation and maturation of dendritic cells (DCs) and other antigen-presenting cells (APCs) in vivo. The results indicated that nanoparticles could promote the expansion and differentiation of antigen-specific T cells by activating APCs, which might be crucial for the immune protection mechanism in vivo.

### 4.3. Evaluation of the Immune Efficacy of Nanoparticle CNPB7 during NDV-IBV Vaccination

In order to investigate the immune-inducing effect of the nanoparticle CNPB7, the Hyline brown chickens were immunized with ND-IB double vaccine and CNPB7 at three concentrations. The spleen, as an important immune organ, is known as the residence of T of B lymphocytes, which play an indispensable role during the immune response process [38]. Therefore, the proliferation level of splenocytes under in vivo stimulation is an important indicator for evaluating the efficacy of immune adjuvants. In this paper, the viabilities of spleen lymphocytes in chicken immunized with vaccine and 100 and 200 μg/mL nanoparticle CNPB7 groups were significantly higher than that in the live vaccine group. It is reported that LPS induces B cell proliferation [39]. As is well known, B cell viability is an important indicator for measuring the humoral immune response in vivo promoted by immune enhancers and vaccines. Therefore, in this manuscript, LPS was used for stimulation in splenocyte viabilities. These results indicated that the combined application of nanoparticle CNPB7 as adjuvants could stimulate the stronger viabilities of lymphocytes in the immunized chicken, which might be vital to the effective immune response to vaccine immunization and antiviral defense mechanism.

The mucosal immune function of chicken is important to the early viral infection and vaccine immunization of NDV and IBV [40,41]. The secreted immunoglobulin A (sIgA) is the primary immunoglobulin involved in mucosal humoral immune response [42]. In this paper, it was observed that the levels of sIgA in the chicken respiratory and digestive tract from the immunized chicken with nanoparticle CNPB7 and NDV-IBV vaccine were significantly increased. These results suggested that the increased sIgA might activate the mucosal immune system of the chicken immunized with nanoparticle CNPB7, and enhance the immune response of T and B cells in submucosal lymphoid tissue. Furthermore, the increase sIgA expression reflects the enhanced mucosal protective ability, which is particularly important in preventing diseases transmitted through mucosal pathways.

Cytokines regulate mucosal function at multiple levels and have a dual effect in maintaining physiological balance and inflammatory response [43]. In this paper, the levels of IL-4, IFN–γ, and IL-13 were increased following nanoparticle CNPB7 vaccination. IL-4, as a cytokine secreted by activated T cells, plays a crucial role in the proliferation and differentiation of B cells [44]. IL-13 is also a cytokine produced by Th2 cells, which has a close functional relationship with IL-4 and plays an important role in regulating immune response, promoting antibody production (especially IgE), and regulating inflammatory response [45]. The increased levels of IL-4 and IL-13 in chicken immunized with nanoparticle CNPB7 indicated that nanoparticle CNPB7 might successfully activate Th2 type immune response, thereby enhancing the B cell-mediated specific antibodies response targeting specific antigens.

Meanwhile, the increase in IFN-γ production was observed in the mucus samples of the respiratory and digestive tract from the immunized chicken. IFN-γ is an important cytokine secreted by specific types of T cells (such as Th1 cells), natural killer cells (NK cells), and NKT cells after recognizing antigens [46,47], which plays a crucial role in regulating immune responses and enhancing the bactericidal ability of macrophages, and regulating the production and activity of other cytokines. These results suggested that nanoparticle CNPB7 might enhance the efficacy of specific vaccines through certain mechanisms, which is vital in infection or disease models that require strong cell-mediated immune responses [48].

The function of nanoparticle CNPB7 on the enhancement of antigen-specific immune responses is very important for the clinical immune application of nanoparticle adjuvants. In this paper, the NDV-IBV dual live vaccine was used as the vaccine model, and the specific antibody response against NDV and IBV was detected. Viral-neutralizing antibodies have the ability to reduce or prevent viral replication [49]. The production of neutralizing antibodies usually involves the activation and differentiation of B cells, which rely on the assistance of T helper cells (CD4^+^T cells) [50]. We found that the neutralizing antibody levels of NDV in chickens immunized with nanoparticles as immune adjuvants CNPB7 were higher than those in vaccine group. Furthermore, it was observed that the titers of antibodies in the chicken immunized with nanoparticle CNPB7 were increased at two weeks after immunization, compared to that of the vaccine control, in which 100 μg nanoparticle CNPB7 stimulated the most significant antibody response. These results indicated that nanoparticle CNPB7 might successfully enhance the humoral immune response to the specific antigen in vaccines, leading to efficient antibody production.

Therefore, the specific increased antibody levels directly reflect the enhanced humoral immune response induced by nanoparticle adjuvant, which is crucial for preventing virus infection and transmission [51]. This immune enhancement function is particularly significant in the development of vaccines adjuvant that require long-term protection or targeting viruses primarily transmitted through mucosal pathways, such as NDV, IBV, and influenza viruses.

## 5. Conclusions

In summary, the safe, stable, and non-cytotoxicity nanoparticle CNPB7 was successfully assembled with bursal-derived peptide BP7, β-glucan, and hyaluronic acid. Our findings demonstrate that the immunization of nanoparticle CNPB7 significantly stimulated the non-specific immune through the upregulation of cytokine expression. Furthermore, CNPB7 effectively promotes the mucosal immune response and antibody production against NDV and IBV. This study provided the experimental basis for the development of a new adjuvant, and supplied the technical support for improving strategies for preventing virus transmission of avian respiratory diseases.

## Figures and Tables

**Table 1:** RT-PCR primer sequence.

Primers	Sequences 5′-3′
CD3-F	GGACGCTCCCACCATATCAG
CD3-R	AAGCTCGTGACATGAGTCCC
CD4-F	TGTGGAACTGTCACCTCGTG
CD4-R	CACATGCATGCAAGGCCAAT
CD80-F	TGTGACCCTCTTTGTCACCG
CD80-R	GGAATCCACGGATTTCGGGT
CD86-F	ACCAGCAAGCTGAATATCCCA
CD86-R	GACTAGCGGCACTGAGACAA
IL-13-F	GCTGGACAACATGACCGACTG
IL-13-R	GCAAGAAGTTCCGCAGGTAGATC
β-actin-F	GAGAAATTGT GCGTGACATCA
β-actin-R	CCTGAACCTCTCATTGCCA

**Table 2:** Reaction procedure of qPCR with the involved primers.

Procedure	Conditions
Pre denaturation	95 °C, 30 S
Cycle condition	95 °C 5 S, 60 °C 20 S, 40 cycles
Melt curve	95 °C 15 S, 60 °C 60 S, 95 °C 0 S

**Table 3:** The optimal parameter screening of composite nanoparticles CNPB7.

NAOH (%)	Sodium Tripolyphosphat (%)	Tween 20 (%)	Tween 80 (%)	Magnetic Stirring Time (h)	Particle Size (nm)	PDI	ZP (mV)
1	0	1	0	2	952.9	0.825	−13.3
1	0	1	0	4	643.9	0.608	−14.4
0	1	0	1	2	643.2	0.547	−19.2
0	1	2	0	4	545.4	0.488	−16.9
0	1	0	1	4	514.9	0.298	−26.5

## Data Availability

The data presented in this study are available upon request from the corresponding author.
